# Advances in gastrointestinal surgical endoscopy

**DOI:** 10.1016/j.amsu.2021.103041

**Published:** 2021-11-17

**Authors:** Reno Rudiman

**Affiliations:** Division of Digestive Surgery, Department of General Surgery, School of Medicine, Padjadjaran University, Hasan Sadikin General Hospital, Bandung, Indonesia

**Keywords:** Current update, Flexible endoscopy, History, Surgical endoscopy

## Abstract

Surgeons have a role in observing, detect abnormalities, disease, and other deficiencies in function which could be treated. Diagnosing and treating back days were challenging for many reasons. However, technology's innovation enhances surgeons' ability to treat their patients. The term *endoscopy* refers to the Greek prefix *endo-* (“within”) and the verb *skopein* (“to view or observe”). Endoscopy is practical both in the diagnosis and treatment of various pathologies. Technological advances, especially in endoscopy, gradually progress and discover many possibilities which allow rapid advancement. Endoscopy development aims to assess human orifice that has not been inspected, probed, and examined over the centuries. Endoscopy over these decades is improving, which led to new problem solving using advanced technological approaches. Thus, a surgeon can solve any issues from examination, diagnosis, and treatment using progressive endoscopy evolution. This review delivers a brief history of advances in surgical endoscopy and describes current endoscopy development.

## Highlights

• The advancement of endoscopies are the breakthrough to the upcoming possibilities in treating patients.
• Each step ahead in various approaches of endoscopy gain further improvement of patient outcomes.
• Diagnostic endoscopy is shifting as a new therapeutic modality to resolve patient's issues.

## 1 History

**Fig. 1:**
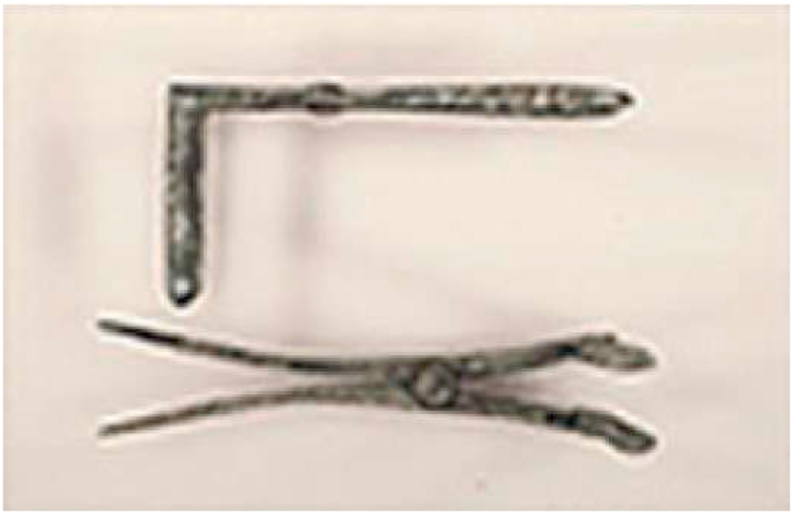
Rectal speculum used by Hippocrates [5]. [Source: Historical Collections & Services, The Claude Moore Health Sciences Library, University of Virginia).

The earliest use of endoscopy was by Hippocrates (460––375 BC). It was used to observe the rectal fistula by using a rectal speculum (Fig. 1) [[1], [2], [3], [4], [5]]. The issue faced by this instrument was inadequate light and shallow depth of penetration; thus, Roman medicine produced a similar device and a three-bladed vaginal speculum discovered in the ruins of Pompeii (AD 70) [3].

**Fig. 2:**
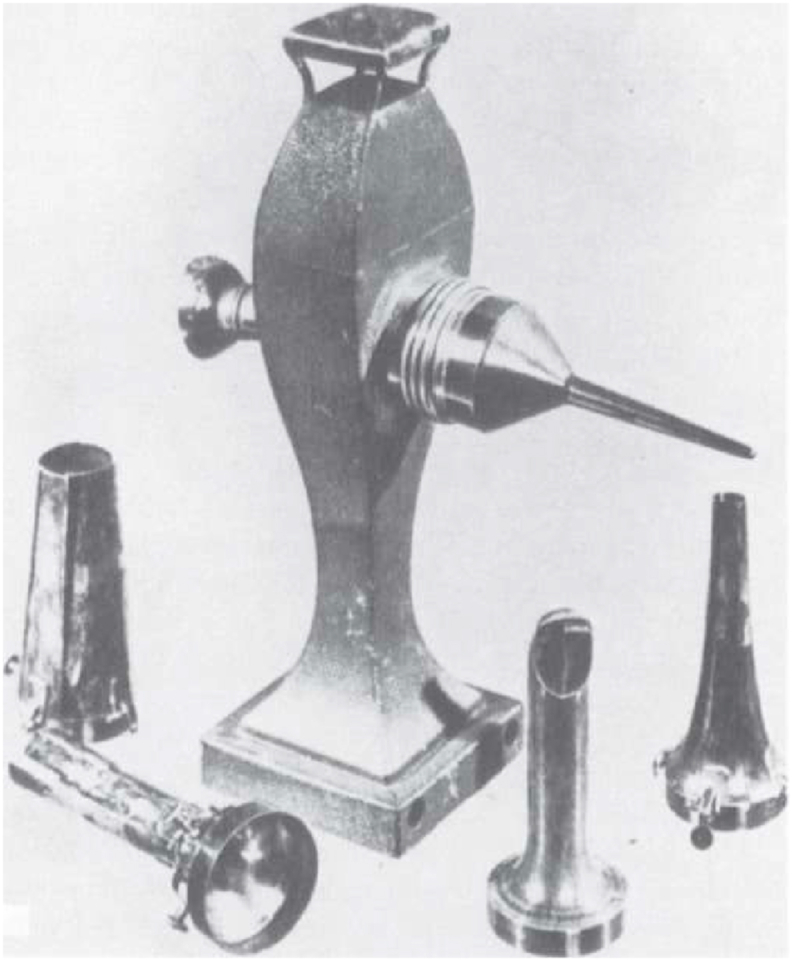
*lichtleiter* [6] (Source: I. H. [Isaac Hayes], Instruments for illuminating dark cavities, *Philadelphia Journal of the Medical and Physical Sciences 14* (1827):410; and Ernest Desnos, *L'Histoire de I'urologie* (1921), 285, Fig. 190).

**Fig. 3:**
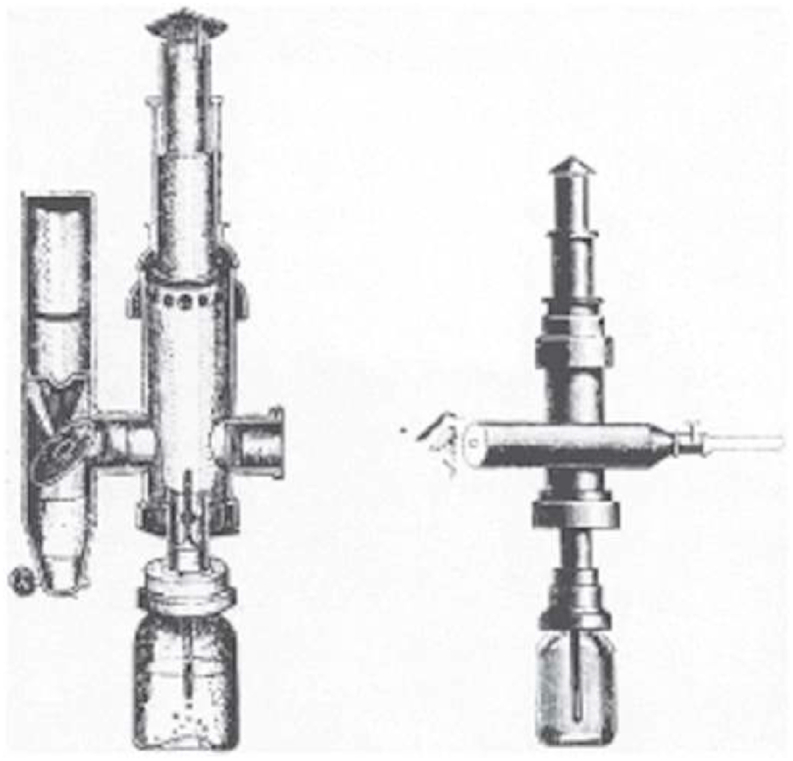
Desormeaux's Endoscope [6]. (Source: Top, J. H. Gemrig, Illustrated catalogue of surgical instruments (ca. 1870), pI. xxx. Bottom, Robert Newman, the endoscope considered particularty in reference to diseases of the female bladder and urethra, Transactions of the Medical Society of the State of New York for the Year 1870, Fig. 2).

Philipp Bozzini (1773–1809) deserved the most credit as the pioneer of modern endoscopy [2,3]. The Bozzini endoscopy is called *lichtleiter* (light conductor), a tin tube illuminated by a candle and reflected using an angled mirror (Fig. 2) [6]. This device was able to exam the urethra, bladder, and vagina [2,7]. In 1826, Pierre Ségalas applied the *lichtleiter* principle that the light reflected by a funnel made of polished silver. Therefore, he called it *the speculum urethro-cystique* [3,5]. Other development was done by Desormeaux in 1855 (Fig. 3); it was a better device although inadequate, even though light source came from lamp fueled with alcohol and turpentine [2,6].

Significant development of endoscopy is originated by Maximilian Nitze (1848–1906), a German urologist who collaborated with Wilhelm Deicke and Louis Beneche to produce a miniature telescope that magnified the image of the bladder using water-cooled platinum wire. Later in 1880, Thomas A. Edison's invention of light was being used by Maximilian to place a small lamp at the end of a cystoscope [5].

**Fig. 4:**
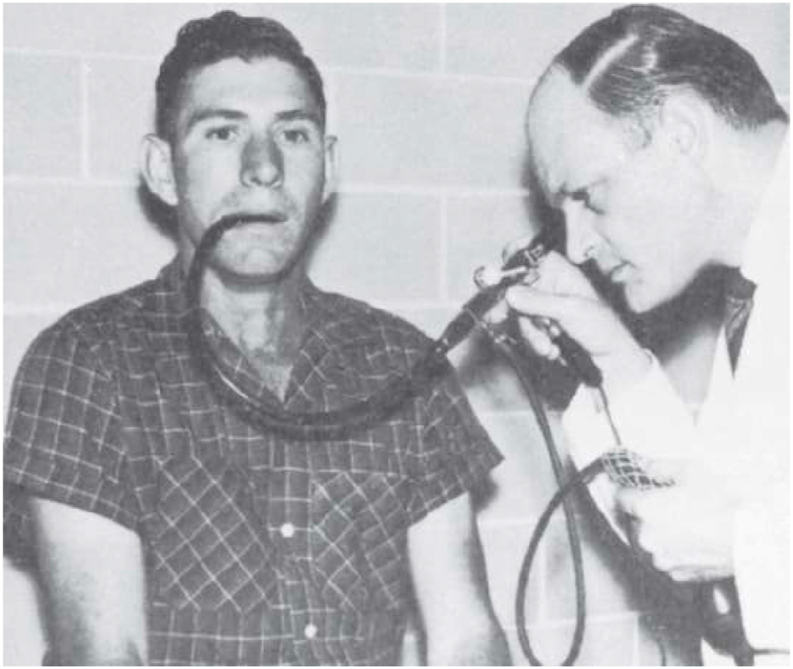
Hirschowitz's fiberoptic endoscope [6]. (Source: Basil I. Hirschowitz, Endoscopic examination of the stomach and duodenal cap with the fiberscope, Lancet 1 (1961):1075, Fig. 2).

The semi-flexible tube endoscope, firstly developed by Georg Kelling in 1898, a surgeon, performed peritoneoscopy by placing a camera at the endoscope's tip. Later, it was improved by Rudolf Schindler in collaborated with Georg Wolf and considered as the “father of gastroscopy.” In 1957, Basil Hirschowitz produced a glass fiber gastroscope and upgraded it into a bundle of fiber called fiberoptic endoscopy (Table 1 & Fig. 4) [3].

**Table 1:** Three periods in the history of gastrointestinal endoscopy [1].

Type	Period
**Rigid Endoscopy**	1805–1932
**Semi-flexible Endoscopy**	1932–1957
**Fiberoptic Endoscopy**	1957 - later

## 2 Types of endoscopy

### 2.1 Per-oral endoscopic myotomy

**Fig. 5:**
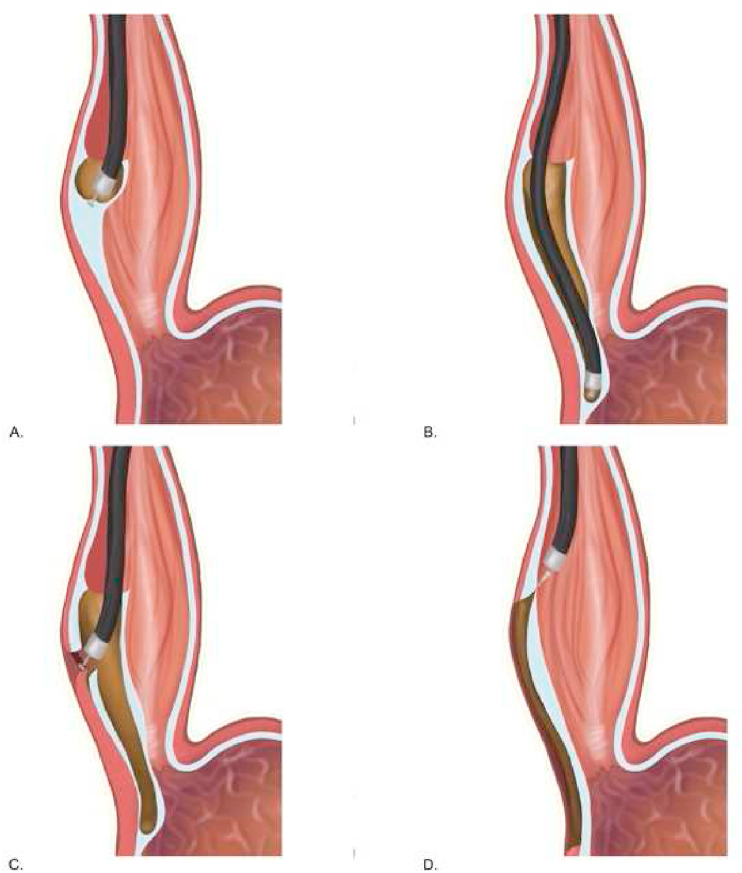
Schematic procedure of POEM. (A) Entry to the submucosal space. (B) Dissecting along with the muscular layer beyond the gastroesophageal junction. (C) Myotomy of the circular esophageal and gastric muscle. (D) Closure of the mucosal entry site.

The first endoscopic myotomy for achalasia was performed in 1980 by Ortega et al. For decades, this technique wasn't well improved, and there was no further report of the procedure. For the first time in Japan in 2008, Inoue et al. performed Per-Oral Endoscopic Myotomy (POEM) in a human being [[8], [9], [10], [11]]. This technique is quite a novel minimally invasive, inspired by the concept of Natural Orifice Transluminal Endoscopic Surgery (NOTES) [9,12]. Initially, POEM was indicated only for nonsigmoid achalasia and later expanded to sigmoid achalasia following the successful procedure in the first five patients (Fig. 5) [8]. The alternative approach to treat achalasia is Heller myotomy; however, this technique is quite more invasive and has prominent adverse effects Table 2 [12].

**Table 2:** Advantages and disadvantages of each technique.

Types of Endoscopy	Year	Developer	Advantages	Disadvantages
**Per-Oral Endoscopic Myotomy**	2008	Inoue et al.	Less invasive, short- to long-term symptomatic relief, cheaper, shorter in duration	Higher complications rate, requires experienced endoscopists
**Percutaneous Endoscopic Gastrostomy**	1980	Gauderer et al.	Long-term use up to 12–18 months, minimal complications, lower mortality rate	Higher cost, requires complicated instrumentations
**Endoscopic Retrograde Cholangiopancreatography**	1968	Dr. William S. McCune	Acts as diagnostic & therapeutic modality, low complications rate	Invasive, limitation of visualization in the proximal ducts, operator dependent, requires sedation
**Endoscopic Ultrasound**	1980	Dimagno et al.	Low adverse events, high resolution imaging, less invasive, no radiation	Operator dependent, higher cost
**Endoscopic Mucosal Resection**	1955	Rosenberg	Noninvasive, lower cost, decreased hospitalization stay and procedural time	Higher risk of recurrence, no fibrotic lesions resection
**Endoscopic Submucosal Dissection**	1990s	Japan	Noninvasive, lower cost, achieves en bloc resection of lesions	Higher risk of recurrence, higher cost than EMR, longer procedural time
**Colonic Decompression**	1977	Kukora et al.	Safe technique, increase the operative time, decreased mortality	High risk of colonic perforation and fistula
**Natural Orifice Transluminal Endoscopic Surgery**	2003	Kalloo et al.	Lesser infection rate, minimal or no visible scar	Higher reconversion percentage, higher cost, suture problems
**Endoscopic Sleeve Gastroplasty**	2013	Abu Dayyeh et al.	Incision-less technique, safe procedure, no mortality	Micronutrients deficiency, high risk of bleeding & gastric fistula

In an earlier report by Inoue et al. in 2010, POEM was performed in 17 patients with achalasia and manifested significant results. Dysphagia symptoms score decreased from mean 10 to 1.3; *p* < 0.0003 and the resting lower esophageal sphincter (LES) pressure decreased from mean 52.4 to 19.9 mmHg; *p* < 0.0001. The short-term outcome (5 months) of POEM was excellent, but further long-term studies are required [9,10]. Other studies of a series of 500 patients by Inoue et al. reported a successful outcome with adverse events found in 3.2% of patients. Significant reductions 2-month post-POEM in symptoms score (Eckardt score 6.0 ± 3.0 vs 1.0 ± 2.0, *p* < 0.0001) and LES pressure (25.4 ± 17.1 vs 13.4 ± 5.9 mmHg, *p* < 0.0001) were achieved [13]. Akintoye et al. reported a meta-analysis of 36 studies involving 2373 patients with significant results Eckardt score ≤3 was achieved in 98% patients post-surgery (95% confidence interval [CI] 97–100%). The mean Eckardt score decreased 6.9 ± 0.15 preoperatively to 0.77 ± 0.10, 1.0 + 0.10, and 1.0 ± 0.008 within 1, 6 and 12 months of treatment respectively. A mean follow-up (8 months) showed the adverse effects of gastroesophageal reflux, esophagitis on esophagogastroduodenoscopy, and abnormal acid exposure were 8.5% (95 %CI 4.9%–13%), 13% (95 %CI 5.0%–23%), and 47% (95 %CI 21%–74%) respectively [12].

### 2.2 Percutaneous endoscopic gastrostomy

**Fig. 6:**
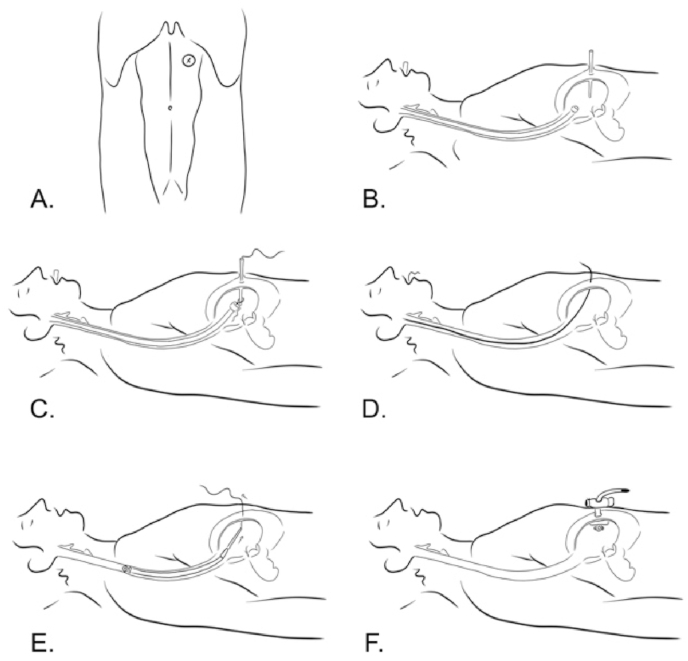
(A) Site of PEG placement. (B) Insertion of the catheter into the gastric cavity. (C) A silk suture is passed through a catheter into the stomach. (D) The suture and gastroscope are removed through the patient's mouth. (E) The mushroom catheter is pulled down to the abdominal wall. (F) A second bumper is positioned in the outer part of the abdominal wall.

Placement of gastrostomy tube percutaneously guided with an endoscope introduced by Gauderer et al. in 1980 (Fig. 6) [[14], [15]]. [[,^15^] Briefly, Percutaneous Endoscopic Gastrostomy (PEG) is a method to place a flexible tube through a temporary tunnel between the abdominal wall and gastric cavity, ensuring a direct passing of food into the patient's digestive tract [16].

This technique was originally developed for children, while nowadays, it is widely used for all ages of patients. Common disease states are responsible for these disorders, such as esophageal cancer, oropharyngeal cancer, esophageal dysmotility, and neurologic conditions (cerebral vascular accident or amyotrophic lateral sclerosis) that impairs or weakens swallowing [17]. Roughly, the two main indications of PEG insertion are enteral feeding and abdominal decompression [18]. Compared to a PEG tube, a nasogastric tube (NGT) results in additional complications, discomfort, and lower feeding efficacy. The advantages of PEG are the long-term use, up to 12–18 months with proper care, and minimal complications [16,18,19].

A review of 150 cases by Ponsky et al. described complications in 15 patients (10%). Superficial wound infections around the catheter were found in 7 patients. Nonetheless, administrating a single preoperative dose of cephalosporin successfully prevented wound infections in 125 cases. Altogether of 150 PEGs, there were 0% mortality and 10% morbidity rates [20]. Another study by Miller et al. with 330 PEGs procedure obtained major complications in 2.1% patients, including five who developed peritonitis. However, no infection occurred at the gastrostomy site and the mortality rate was 0.6% [21]. Therefore, optimizing post-surgical care, preventive strategies, and treating early complications will maximize safety and effectiveness outcomes [22].

### 2.3 Endoscopic retrograde cholangiopancreatography

**Fig. 7:**
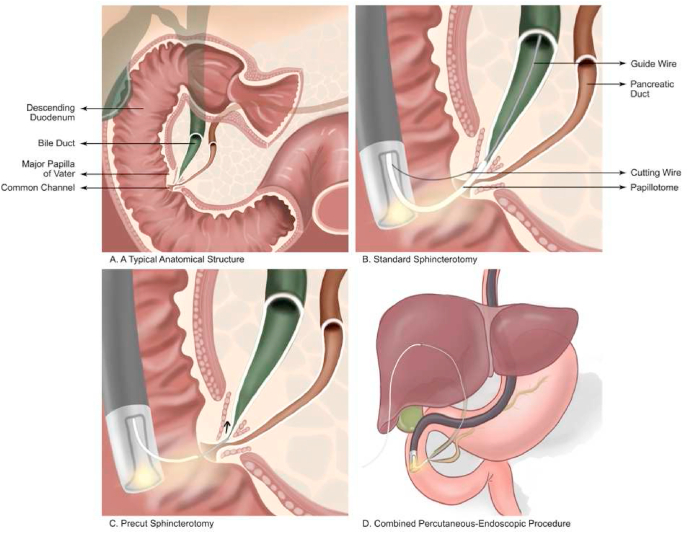
(A) A typical anatomical structure, (B) Endoscopic biliary sphincterotomy, (C) A needle-knife precut method, (D) A transhepatic antegrade passage of a guidewire for completing the sphincterotomy.

Endoscopic Retrograde Cholangiopancreatography (ERCP) was introduced in 1968 by Dr. William S. McCune, an obstetrician who performed ERCP using a fiber duodenoscope for a diagnostic tool. Later in 1972, Dr. Peter Cotton introduced cannulation in ERCP. In the following years, Dr. Meinhard Classen in Germany and Keiichi Kawai in Japan discovered a therapeutical potential of ERCP with endoscopic sphincterotomy [[23], [24], [25]]. Briefly, ERCP (Fig. 7) combines endoscopy and fluoroscopy to treat pancreaticobiliary diseases. An endoscope is inserted until the ampulla of Vater is identified. A guidewire is then passed through the endoscope into the biliary or pancreatic ducts through the ampulla of Vater; this step is referred to as cannulation. Later, injection of contrast medium is performed under fluoroscopy to visualize the anatomy of biliary and pancreatic ducts [26].

The advances of high-resolution imaging modalities such as magnetic resonance imaging with magnetic resonance cholangiopancreatography (MRCP), pancreatic protocol computed tomography scans, endoscopic ultrasound (EUS) have substituted ERCP as a diagnostic tool. Thus, the therapeutic/interventional approach becomes the main focus of ERCP [[25], [26], [27]].

The indications of ERCP may vary in clinical situations. Most commonly is used to treat biliary problems (choledocholithiasis, ascending cholangitis, strictures, biliary stenting, etc.) than pancreatic issues such as pancreatitis, pancreatic fistule, pancreatic fluid collections, etc. [26,28] Endoscopic retrograde cholangiopancreatography is contraindicated in uncooperative patients, bowel perforation, esophageal stenosis, coagulopathy and inability to sedate [26].

Various complications of ERCP might occur, such as post-ERCP pancreatitis (PEP), hemorrhage, perforation, cholangitis, cholecystitis, cardio-pulmonary depression, asymptomatic hyperamylasemia, aspiration, bleeding, hypoxia, sepsis, and death [24,27,28]. In a prospective, 2-year study of 2,347 patients from 17 institutions reported the most common complications, for example, PEP (9.8%), pancreatitis (5.4%), and hemorrhage (2%) [29]. Wang et al. performed 3,178 ERCP procedures in 2,691 patients and overall complications were developed in 213 (7.92%) patients, pancreatitis in 116 (4.31%), and asymptomatic hyperamylasemia in 396 (14.72%) [30]. Other report in the United Kingdom (UK) with data on 5264 ERCP, 230 patients (5%) suffered multiple complications such as pancreatitis in 74 (1.6%), cholangitis in 48 (1%), hemorrhage in 40 (0.9%), perforation in 20 (0.4%), and miscellaneous in 54 (1.2%) [31]. Study from Andriulli et al., involving 16,855 patients, ERCP-attributable complications are 1,154 (6.85%) with the following pancreatitis occurred in 585 patients (3.47%), infections in 242 (1.44%), bleeding in 216 (1.34%) and perforations in 101 (0.60%) [32].

### 2.4 Endoscopic ultrasound

Endoscopic ultrasound (EUS) has advanced from a diagnostic imaging modality discovered by Dimagno et al. in the 1980s to an interventional procedure [[33], [34], [35], [36]]. The advantage of EUS is to visualize, interrogate and intervene gastrointestinal (GI) luminal, mural, or peri-mural structures and pathologies with minimal adverse events [37,38].

**Fig. 8:**
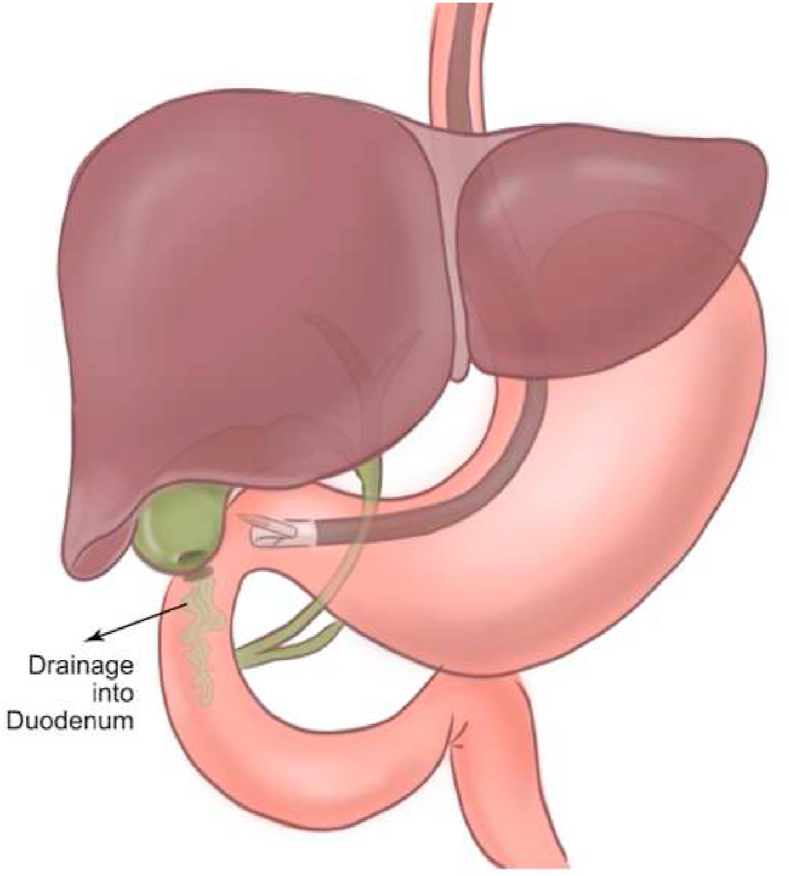
Interventional EUS-guided gallbladder drainage through the duodenal wall.

Diagnostic EUS uses an echo-endoscope with several variants, such as radial or linear, and developed in 1991, referred to as EUS-fine-needle aspiration (EUS-FNA) [37]. On the other hand, interventional EUS has evolved with many techniques, for example, EUS-guided drain-age (GD) of pancreatic fluids (PFCs), EUS-guided necrosectomy, EUS-guided cholangiography and biliary drainage (BD) (Fig. 8), EUS-guided pancreatography and pancreatic duct drainage (PDD), EUS-guided gallbladder drainage, EUS-GD of abdominal and pelvic fluid collections, EUS-guided celiac plexus block (CPB) and celiac plexus neurolysis (CPN), EUS-guided pancreatic cyst ablation, EUS-guided delivery of anti-tumoral agents and EUS-guided fiducial placement, brachytherapy and EUS-guided vascular interventions [34,[38], [39], [40]]. Most current literature suggests both diagnostic and interventional EUS as a feasible, safe, efficacious, and less invasive modality [37,41].

Current EUS with radial and linear echo-endoscope has a non-flexible transducer which produces a more rigid tip, 3–5 cm length, of echo-endoscope. Furthermore, an oblique endoscopic view, the echo-endoscope insertion, and advancements of the instruments create a semi-manual maneuver; thus, adverse events, though rare, include perforation, bleeding, and infection. A study involving 43,852 subjects reported only 16 (0.03%) cervical esophageal perforation with only one death within the EUS procedures [37]. A prospective study by Bournet et al. reported zero death, no surgery, and three mild complications among patients who did diagnostic EUS. There were also five complications in interventional EUS, such as acute pancreatitis, duodenal perforation, upper digestive bleeding, and mediastinal infection with a mean delay of the occurrence is 30 h, and the mean duration of hospitalization is 7 days [42]. In comparison with diagnostic EUS, interventional EUS has a higher risk of complications due to operator-based experience and procedure difficulty. Nevertheless, interventional EUS has an overall success rate of more than 90% [43].

### 2.5 Endoscopic mucosal resection and endoscopic submucosal dissection

The history of endoscopic resection began in 1955 when Rosenberg introduced endoscopic mucosal resection (EMR) by creating a plane for fulguration of sigmoid and rectal polyps. Then in 1973, Dehyle utilized submucosal injection to the sessile or flat lesions for complete removal. At first, EMR was unpopular and regarded as a risky procedure. In the meantime, it became accepted in 1980, but sometimes EMR isn't dependable to ensure the complete resection of the tumors. Hence, endoscopic submucosal dissection (ESD) was developed in the 1990s for *en bloc* resection of lesions [[44], [45], [46], [47], [85]].

**Fig. 9:**
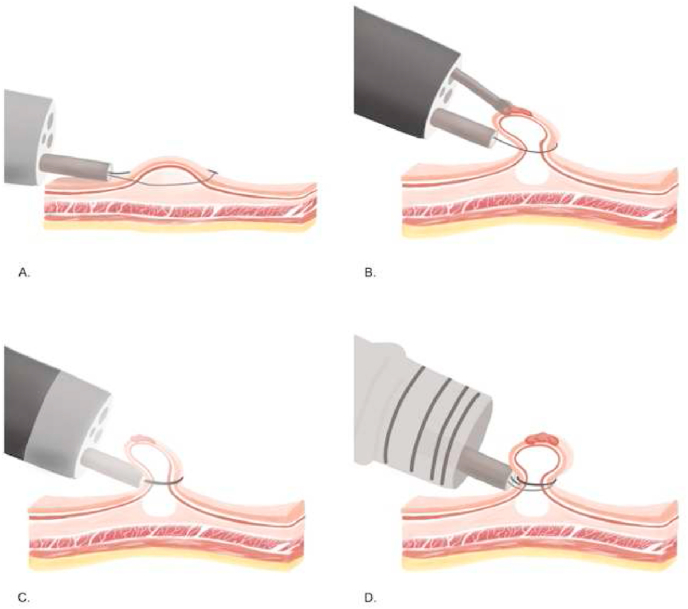
Four techniques of EMR. (A) The Inject-and-cut technique. (B) The Inject-lift-and-cut technique. (C) The cap assisted EMR technique. (D) The ligation-assisted technique.

**Fig. 10:**
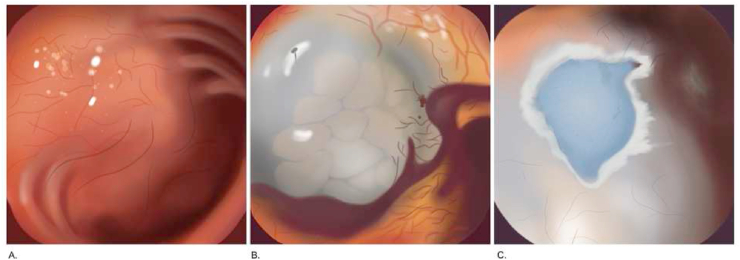
Endoscopic mucosal resection. (A) Identification of flat lesion. (B) Injection of saline-tinted methylene blue dye solution. (C) Lesion appearance after EMR procedure and the muscular propia layer tinted with methylene blue dye. (For interpretation of the references to colour in this figure legend, the reader is referred to the Web version of this article.)

**Fig. 11:**
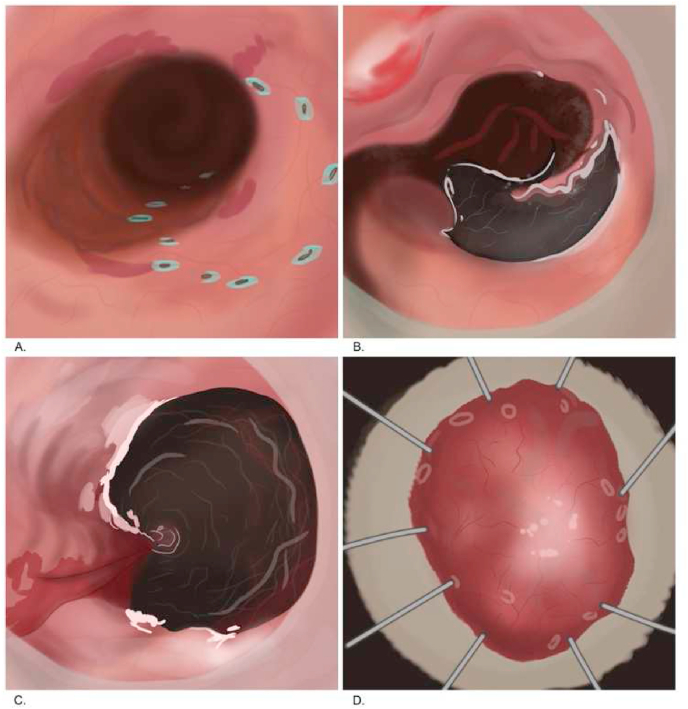
Endoscopic submucosal dissection in early esophageal adenocarcinoma. (A) Marking. (B) Partial circumferential incision. (C) ESD mucosal defect. (D) Resected specimen.

Endoscopic mucosal resection is a technique for removing sessile or flat lesions to the superficial layers of the gastrointestinal (GI) tract (Fig. 10). The maximum lesion diameter for resection is around 20 mm due to the physical size limitation of the operating snare. Therefore, lesions above 20 mm were removed by piecemeal resection with a higher chance of recurrence. The commonly used techniques are injection-, cap-, and ligation-assisted EMR (Fig. 9) [44,45,[48], [49], [50], [86], [87]]. The recent development is underwater EMR for salvaging EMR [51]. Injected-assisted EMR starts with injecting a solution into submucosal space, creating a “safety cushion.” Then the lesions are easily removed and minimize damage to gastrointestinal walls. This method can be further divided into the “inject-and-cut” technique and the “inject-lift-and-cut” technique [49]. The cap-assisted EMR requires a transparent plastic cup at the distal tip of the endoscope. The lesion is sucked into the cap, and the snare is closed at the base of the lesion. Otherwise, in the ligation-assisted EMR, a rubber band is deployed at the base to create pseudo-polyp [46]. Endoscopic submucosal dissections developed for *en bloc* removal of large tumor above 20 mm and flat GI lesion (Fig. 11). Normal saline or sodium hyaluronate is injected into the submucosa layer of the lesion. The fluid-expanded submucosal space creates a precise resection control. Thus, achieving a radical excision of the lesions [[45], [51]]. [[45], [51]].

Both EMR and ESD are required for definitive therapy of early-stage (T1mN0) and malignant lesions of the GI tract. Another function of EMR and ESR is to obtain larger histological specimens and provide an accurate histologic T staging [45,49]. The major advantages of EMR are the relatively short time of the procedure (approximately 35 min for larger lesions), lower bleeding risk in 0.9%, and low perforation rate between 0.4 and 1.3% [52,53]. Fukami et al. also reported a higher complication rate in ESD with a bleeding risk of 4.8–5.7% compared with 2.3–3.5% in EMR. Perforation risk is 4.8% in ESD compared with 0.9–1.4% in EMR [54]. Meanwhile, ESD is superior in the *en bloc* resection, the complete resection, and the recurrence rates based on Lee et al. comparing EMR and ESR with a result of 42.9% vs 92.7%, 32.9% vs 87.6%, and 25.9% vs 0.8%, respectively [50]. Therefore, ESD is relatively superior to EMR and may prevent unnecessary surgery. Nevertheless, ESD requires highly trained operators and intensive training to reduce iatrogenic adverse events [46].

### 2.6 Colonic decompression

Acute colonic, such as acute colonic pseudo-obstruction (ACPO), colonic volvulus, and malignant obstruction, is a medical emergency with high morbidity and mortality. Colonic decompression is one of the established treatment strategies [55]. The first colonic decompression in 1977 by Kukora et al. with successful colonoscopic decompression in six patients for ACPO [[56], [57], [58]]. Following years later, Bernton et al. developed a new technique by including transanal colonic tubes with outstanding results [57]. In 2002, Morino et al. proposed a new minimally invasive procedure called endoscopic stent decompression. The result was quite promising, with zero complications [59]. Mainly, the goals of colonic decompression are to reduce the colon diameter, reduce wall tension, allow the blood to circulate, and restart peristaltic movement [55].

The advancing methods of colonic decompression expand the choices included radiologic placement of decompression tubes under fluoroscopy guidance and colonoscopic decompression with or without placement of a decompression tube. These techniques are recommended by the European Society of Gastrointestinal Endoscopy (ESGE) if the cecal diameter is more significant than 12 cm [55,60]. For performing colonic decompression, an endoscope is inserted through the site of obstruction. The obstructed lumen is identified by a black hole or tiny gas bubbles escaping from the block. A flexible guidewire is pushed through beyond the obstructed site. The endoscope is withdrawn, and a lubricated tube is advanced through the guidewire. The indications of successful insertion are immediate escape of air and liquid feces through the catheter [61].

**Fig. 12:**
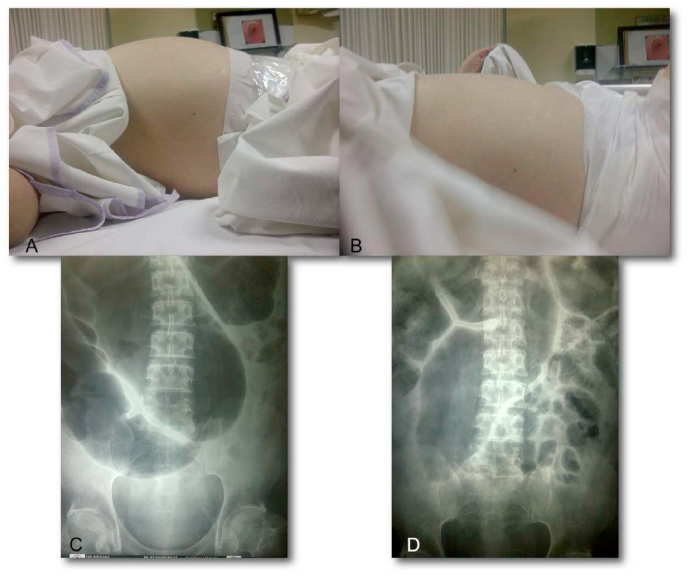
(A) Clinical condition before ileus tube placement. (B) Seven days after colonic decompression. (C&D) Plain abdominal X-ray of the distended large bowel and an air-fluid level.

A retrospective cohort study of 53 patients reported a clinical success rate of up to 92.5% and an additional decompression tube at an 86% success rate. The complication rate was 3.8% with one perforation [55]. Bode et al. described a series of 22 patients of ACPO who underwent colonic decompression. They summarized a 91% success rate in 20 of 22 patients, and a 4.5% complication rate resulted in the death of one patient [58]. Fischer et al. also narrated a promising result of successful endoscopic tube placement for 43 of 51 patients (84%) [62]. A comparative study of the standard medical therapy and colonic decompression groups included 61 and 83 patients who stated superiority of colonic decompression in several aspects, including complete resolution rate, readmission rate, and mortality, with overall results 19.9% vs 47.7%, 26.2% vs 15.7%, and 14.8% vs 8.4%, respectively [63]. In China, colonic decompression (Fig. 12) using the ileus tube was successfully performed in 45 of 46 patients (97.8%), with no leakage or stenosis occurred postoperatively [64]. All these studies stated above concluded the colonic decompression with or without decompression tube have proved to be safe, effective, and highly successful for treating colonic distention [55,58,[62], [63], [64]].

### 2.7 Natural orifice transluminal endoscopic surgery

Natural orifice transluminal endoscopic surgery (NOTES), widely known as a “scarless surgery,” has withdrawn attention in these recent years as a novel surgical method to develop minimally invasive surgery [65,66]. Kalloo et al. [67] is the pioneer of NOTES performed on swine. Later, the first human *trans*-gastric NOTES appendectomy was demonstrated by Rao and Reddy [68]. Originally, NOTES was used for diagnosing and treating abdominal abnormalities, then improved to perform any surgery through natural orifices such as *trans*-esophageal, *trans*-gastric, *trans*-vaginal, *trans*-vesical, and *trans*-colonic. The main aims of the NOTES technique are to minimalize cosmetic alteration, avoid abdominal incisions, and reduce invasiveness compared to traditional laparotomy or laparoscopic surgery [[69], [70], [71]].

**Fig. 13:**
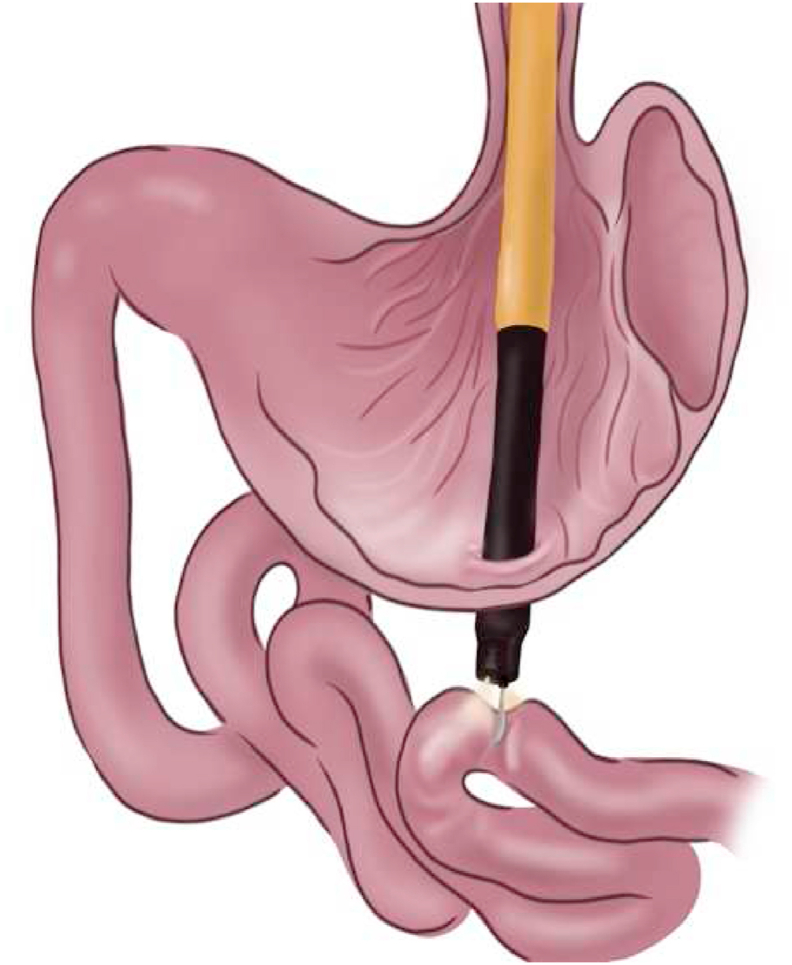
Schematic view of endoscopic forceps grasps small bowel.

**Fig. 14:**
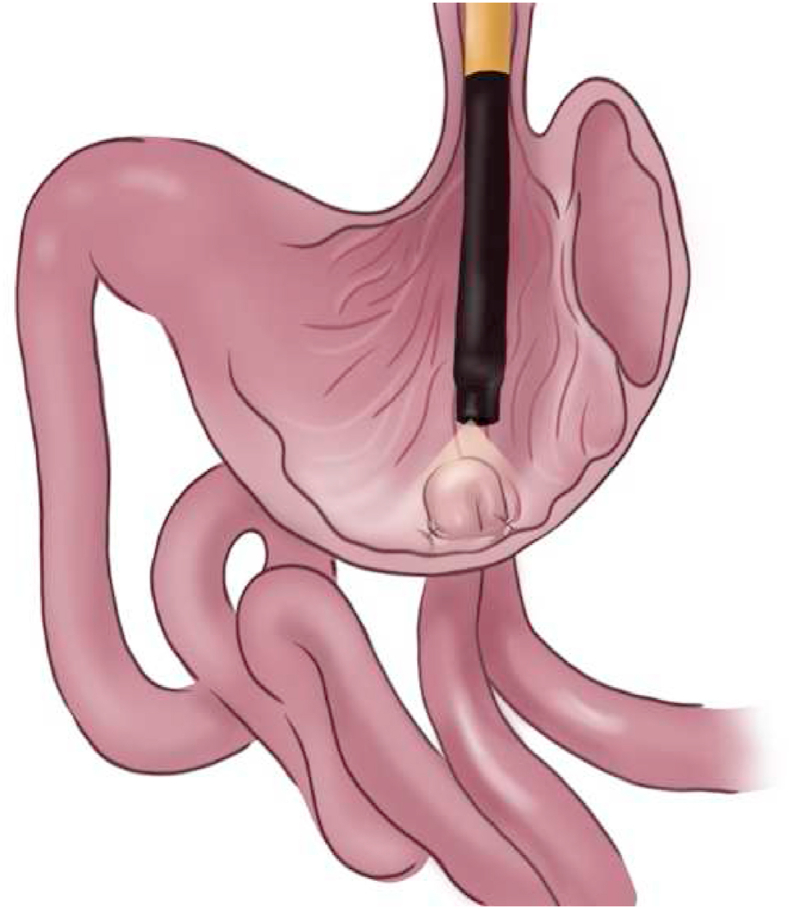
Schematic view of small bowel fixed inside the gastric cavity with incisions.

The procedure begins with a standard single-channel gastroscopy and placement of an overtube. Clearance of the gastric contents by suction and gastric lavage, then flush the stomach with an antibiotic. Cefazolin is the recommended option for maximum results. The cleansing step has not been studied; however, this procedure is required to maintain the sterility of the peritoneal cavity. An incision is made in the anterior gastric wall as the most common site. Various instruments, including the needle-knife, insulation tipped (IT) knife, controlled radial expansion (CRE) balloon, or pull-type sphincterotome, can be used for gastrostomy. Subsequently, a gastroscope is inserted into the peritoneal cavity and visualizes abdominal organs (Fig. 13, Fig. 14). Various procedures can be performed with many accessories of the endoscope (e.g., endoloops, endoclips, and biopsy). After the process is complete, the incision is closed with endoclips or suturing device [72].

As a novel method, NOTES provides many advantages compared to traditional laparotomies, such as zero incision and scar, less pain, injury, a reduced dosage of analgesic and anesthetic, rapid recovery, reduce hospitalization, and decrease inpatient complications (nosocomial infection, deep vein thrombosis, and pulmonary embolism) [69,73]. Consequently, NOTES will not accept widespread adoption until several limitations are solved. The endoscope is relatively small, making the retraction and dissection more challenging; hence, larger and solid instruments are necessary. Orientation also becomes a major challenge for NOTES due to the triangulation of laparoscopy is impossible. Some organs are difficult to localize (spleen and gallbladder); thus, EUS or mini probe (MP) aid is required. Closure and suturing are another main focus of NOTES because of the importance of minimizing perforation and infection risk. To date, animal studies of closure of the transluminal access were unsatisfactory due to microabscesses, peritonitis, and death. However, an available, safe, and simple closure instrument has not been found [[74], [88]].

### 2.8 Endoscopic sleeve gastroplasty

Obesity, type 2 diabetes mellitus, and cardiovascular disease dominate the epidemic proportions [75]. Bariatric surgery is a well-approved, proven, and effective solution for obesity and associated comorbidities [76,77]. Fogel et al. [78] in 2008 and Brethauer et al. [79] in 2010 demonstrated endoscopic gastric reduction using a superficial suturing device. Then, Abu Dayyeh et al. [75] performed the first endoscopic sleeve gastroplasty (ESG) by creating a small diameter sleeve along the lesser curvature of the stomach.

**Fig. 15:**
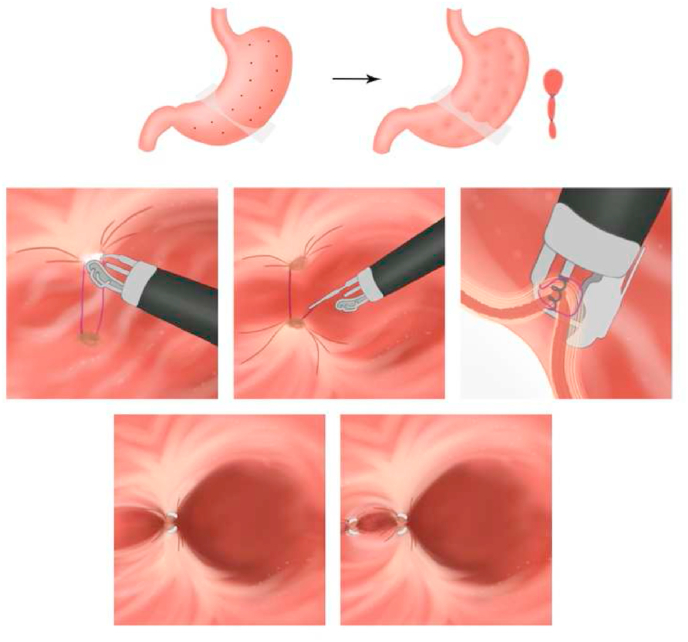
Interrupted triangular suture technique in ESG.

**Fig. 16:**
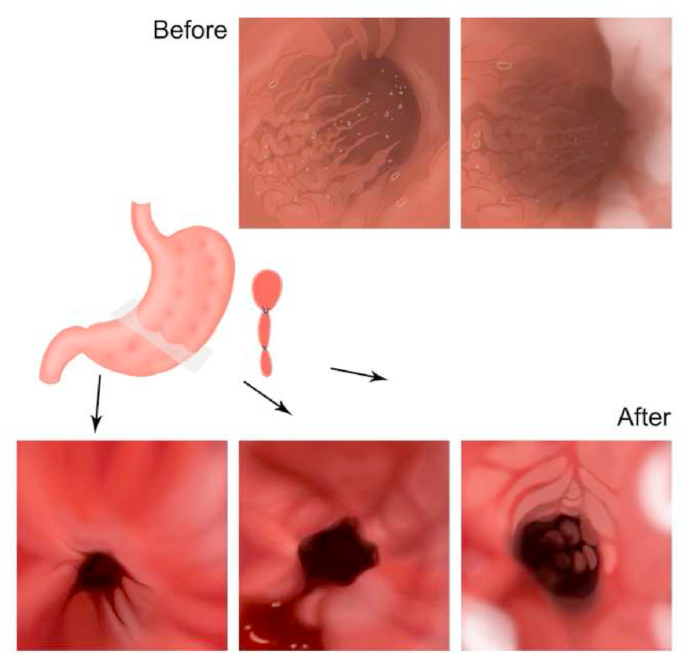
Before and after ESG.

Endoscopic sleeve gastroplasty is an incision-less and minimally invasive technique that intentionally reduces the functional volume of the stomach by 80% using an interrupted triangular suture pattern created along the greater curvature of the stomach (Fig. 15, Fig. 16) [76,80,81]. Many studies proved ESG is an effective and safe method for reducing body weight and associated comorbidities. A prospective study of 91 patients with mean body mass index (BMI) 40.7 ± 7 kg/m^2^ had lost 14.4% of total BMI at the first six months, 17.6% at 12 months, and 20.9% at 24 months, alongside with significant reduction in hemoglobinA1c (*p* = 0.01), systolic blood pressure (*p* = 0.02), waist circumference (*p* < 0.001) and serum triglycerides (*p* = 0.02) [82]. Lopez-Nava et al. reported a mean BMI reduction in 50 patients from 37.7 ± 4.6 to 30.9 ± 5.1 kg/m^2^ at one year with no significant adverse events [83]. Other studies stated post-ESG results in 10 patients with a mean BMI of 45.2 kg/m^2^ after one month, three months, and six months mean weight loss of 11.5 kg, 19.4 kg, and 33 kg no adverse events noted [84]. Thus, ESG is a practical, reproducible, and safe procedure to decrease body weight and prevent further complications due to associated comorbidities.

## 3 Conclusions

Inventions and developments of surgical techniques in endoscopy have evolved within decades. Surgeons collaborate to generate something according to patient conditions to minimize adverse events, enhance life quality, and improve safety. Alongside technology's advancement and surgeons' capability, recent methods are improving and promising a genuine endoscopy; thus, each step ahead alters the function of endoscopy into branches.

## Ethical Approval

Author does not require ethical approval in this article.

## Sources of funding

Author declares no funding of this article.

## Author contribution

Author contributes in study concept, data collection, and writing the paper of this article.

## Consent

Author does not need consent in this article.

## Registration of Research Studies

Author does not need registration of research studies.

## Guarantor

Reno Rudiman acts as the author and the guarantor of this article. Author is fully responsible for the work, the conduct of the study, data accessibility and decision to publish.

## Disclosure

The author reports no conflicts of interest in this work. This paper abstract was presented at the 11th Global Gastroenterologists Meeting named Recent Advances in Surgical Endoscopy as a poster presentation. The poster abstract was published in “Poster Abstracts” in the Journal of Gastrointestinal & Digestive System. DOI: 10.4172/2161-069X–C1-049.

## Declaration of competing interest

Author declares no conflict of interest.
